# Welcome Word Annual Meeting BSR 2023

**DOI:** 10.5334/jbsr.3312

**Published:** 2023-12-08

**Authors:** Piet Vanhoenacker

**Affiliations:** 1 University Hospital Gent, BE

**Keywords:** Welcome Address, Radiology Conference, Medical Imaging

Dear Friends and Colleagues,

Warm welcome to the 2023 Annual Meeting of the Belgian Society of Radiology. It is a distinct pleasure to gather again with everyone dedicated to the ever-evolving realm of radiology.

This year, the focus is on a topic of vital significance: vascular imaging throughout the entire body. The vasculature, intricate and essential, is not just a network of vessels but a testimony to the marvel of human anatomy and physiology. Rapid progress in imaging technologies now enables us to capture and analyze this system with unprecedented detail and reality, leading to improved diagnosis and treatment strategies, as well as, ultimately, improved prognosis and patient outcome.

Given the overwhelming range of topics in this field, it has been challenging to select appropriate lectures for a one-day event. Thanks to the huge efforts of the Scientific Section of Non-Invasive Cardiovascular Imaging and the invaluable contribution from the Young Radiologists Section (YRS), an attractive program has been composed, promising to be both insightful and inspiring.

The goal of the sessions is to share the latest techniques, with innovative applications of vascular imaging. Active participation is encouraged to get the most of it for your routine practice. Indeed, the annual meeting is as much about the exchange of ideas as it is about learning.

I sincerely hope this meeting serves as a fruitful platform for teaching and learning, as well as for networking, and hopefully even sparks the flame of new ideas or collaboration.

Thank you for joining and enjoy an enlightening meeting!

Piet Vanhoenacker

Chair Scientific Board BSR

